# New insight into neurological degeneration: Inflammatory cytokines and blood–brain barrier

**DOI:** 10.3389/fnmol.2022.1013933

**Published:** 2022-10-24

**Authors:** Jie Yang, Mingzi Ran, Hongyu Li, Ye Lin, Kui Ma, Yuguang Yang, Xiaobing Fu, Siming Yang

**Affiliations:** ^1^Research Centre for Tissue Repair and Regeneration Affiliated to the Medical Innovation Research Department, PLA General Hospital, PLA Medical College, Beijing, China; ^2^Department of Dermatology, 4th Medical Centre, PLA General Hospital, Beijing, China; ^3^Department of Anaesthesiology, 4th Medical Centre, PLA General Hospital, Beijing, China; ^4^Department of Neurology, The First Medical Centre, PLA General Hospital, Beijing, China

**Keywords:** inflammatory cytokines, blood-brain barrier, neurological degeneration, TNFα, IL-1β, IL-6

## Abstract

Neurological degeneration after neuroinflammation, such as that resulting from Alzheimer’s disease (AD), stroke, multiple sclerosis (MS), and post-traumatic brain injury (TBI), is typically associated with high mortality and morbidity and with permanent cognitive dysfunction, which places a heavy economic burden on families and society. Diagnosing and curing these diseases in their early stages remains a challenge for clinical investigation and treatment. Recent insight into the onset and progression of these diseases highlights the permeability of the blood–brain barrier (BBB). The primary factor that influences BBB structure and function is inflammation, especially the main cytokines including IL-1β, TNFα, and IL-6, the mechanism on the disruption of which are critical component of the aforementioned diseases. Surprisingly, the main cytokines from systematic inflammation can also induce as much worse as from neurological diseases or injuries do. In this review, we will therefore discuss the physiological structure of BBB, the main cytokines including IL-1β, TNFα, IL-6, and their mechanism on the disruption of BBB and recent research about the main cytokines from systematic inflammation inducing the disruption of BBB and cognitive impairment, and we will eventually discuss the need to prevent the disruption of BBB.

## Introduction

Aging body is vulnerable to chronic inflammatory conditions that cause neurological degeneration in the central nervous system (CNS), such as Alzheimer’s disease (AD) (Ju and Tam, 2022). These diseases are frequently associated with high mortality and morbidity and with permanent cognitive dysfunction, which are commonly considered a significant economic burden on society and on the families of patients. The difficulties faced in resolving these clinical problems are related to the inability of the damaged and degenerated nerve cells to repair themselves (Ren et al., 2018). Although recent research indicates that necrotic foci can be replaced by proliferative neural stem cells (NSCs) (Kuhn and Svendsen, 1999), the number and scale of the NSCs cannot make up for the entire injury to the CNS, and strategies for curing neurological diseases in the clinic remain far off. Interestingly, recent research finds that these diseases share a key pathological feature, namely, the disruption of the blood–brain barrier (BBB) (Sweeney et al., 2019; Gold et al., 2021). The disruption of BBB can be detected before the onset of neurodegeneration, and the repairing of BBB after neurodegeneration can be beneficial for the disease (Liebner et al., 2018). Therefore, studying the biological and pathological features of BBB may be a useful target for future diagnosis and treatment.

A primary cause to disrupt the BBB is uncontrolled inflammation after injuries or diseases. Under this condition, the abnormal increasing pro-inflammatory cytokines such as TNFα (Chen et al., 2019), interleukin-1beta (IL-1β) (Hauptmann et al., 2020), interleukin-6 (IL-6) (Yang et al., 2017; Yang J. et al., 2020), interferon-γ (INF-γ) (Bonney et al., 2019), and inducible nitric oxide synthase (iNOS) (Smith et al., 2018) can compromise BBB permeability and induce or deteriorate neurological disorders which make the regulation of inflammation more difficult (Ren et al., 2020). In addition, although BBB structure becomes compromised with aging, leaving patients vulnerable to neurological diseases such as AD (Huang et al., 2020), the main inducer to disrupt the BBB is the abnormal increasing inflammatory cytokines after injuries or diseases.

Moreover, most research focus only on the primary diseases or injuries of the brain inducing the dysfunction of BBB and thus CNS impairment, but few on the peripheral injuries leading to that. Our previous studies indicated that peripheral burns and traumatic surgical wound could induce the dysfunction of BBB and thus cognitive impairment through IL-6 and IL-1β (Yang et al., 2017; Yang J. et al., 2020). As a result, regulating inflammatory factors from assaulting the BBB is crucial for curing neurological diseases. In this review, we will systematically discuss how these pro-inflammatory cytokines assaulting on BBB.

## The structure and biomechanisms of blood–brain barrier

**FIGURE 1:**
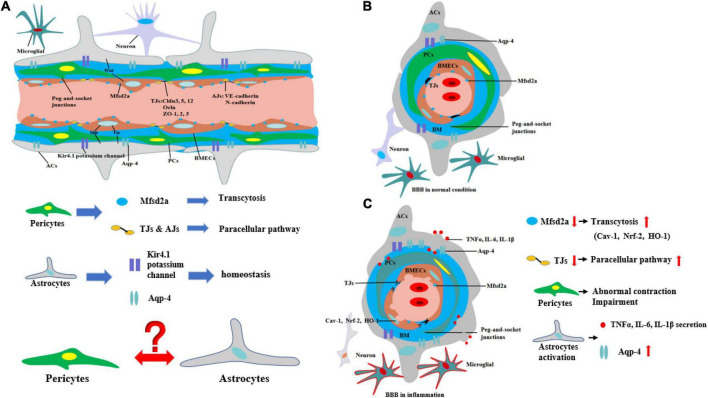
Structure and biomechanisms of blood–brain barrier during physical or inflammation. **(A)** The structure of BBB from longitudinal section during physical condition. PCs control the transcytosis and paracellular pathway of BBB by regulating the expression of Mfsd2a and TJs. The structure of potassium channel of Kir4.1 potassium channel and Aqp-4 on ACs regulates the homeostasis of CNS. However, the relationship between ACs and PCs is still unknown. **(B)** The structure of BBB from cross-section during physical condition. **(C)** The structure of BBB from cross-section during inflammation. During inflammation, the expression of Mfsd2a is decreasing, which is negatively correlated with transcytosis of BBB. The level of TJs is decreasing, which means the paracellular pathway of BBB is increasing. Moreover, PCs are abnormal contracted and impairment. In addition, ACs are activated to secrete pro-inflammatory cytokines and the level of Aqp-4 is increasing, which is correlated with the deterioration of CNS diseases or injuries.

In contrast to the peripheral vasculature, the vessels in the brain possess a highly selectively permeable barrier, BBB, that protects the CNS from potential toxins, pathogens, and so on. Only small lipid-soluble molecules <400 Da and with fewer than nine hydrogen bonds can independently cross the BBB *via* lipid-mediated diffusion (Padden et al., 2007; Segarra et al., 2021). BBB comprises a tightly sealed monolayer of brain endothelial capillaries (Ohtsuki and Terasaki, 2007) containing cell types such as brain microvascular endothelial cells (BMECs), pericytes (PCs), astrocytes (ACs) end feet, microglia, and neurons (Figures 1A,B; Bazzoni and Dejana, 2004). Molecules between the blood circulation and the brain parenchyma through BBB by two ways include transcellular vesicular transport (transcytosis) and paracellular pathway. BMECs are connected to one another by tight junctions (TJs) and adherens junctions (AJs) (Hawkins and Davis, 2005), which regulate the paracellular permeability of BBB (Bazzoni and Dejana, 2004). TJs include the endothelial-specific claudin family member claudin-5 (Cldn5) and occludin (Ocln), which are linked to the cytoskeleton by members of the zonula occludens family (ZO-1, ZO-2, and ZO-3) (Brown and Davis, 2002). The interactions among these proteins are the primary factors regulating the paracellular barrier of BMECs (Sandoval and Witt, 2008; Figure 1A). AJs, which are formed by homophilic interactions between cadherins, such as vascular endothelial (VE)-cadherin (Cdh5, CD144) and N-cadherin, are considered a prerequisite for the establishment of TJs (Figure 1A). In BMECs, AJs intermingle with TJs to form junctional complexes that contribute to BBB stability (Dejana and Orsenigo, 2013). Another way, transcytosis, is mainly regulated by PCs, which are embedded along with vascular mural cells in the basement membrane of brain microvessels and abnormal PC function may lead to BBB dysfunction and neuroinflammation (Daneman et al., 2010; Dohgu and Banks, 2013; Ben-Zvi et al., 2014). Furthermore, recent research indicates that an important membrane protein, major facilitator superfamily domain containing 2a (Mfsd2a), on BMECs also plays an important role on transcytosis, and its expression may be regulated by PCs (Ben-Zvi et al., 2014) (Figures 1A,B). The reduced Mfsd2a after CNS diseases or injuries can increase the levels of transcellular vesicles including Cav-1, Nrf-2, and HO-1 on BMECs which may contain toxins, pathogens, or cytokines in brain, while upregulation of Mfsd2a will protect BBB from decreasing levels of the vesicles (Eser Ocak et al., 2020; Figure 1C).

Moreover, ACs are also the principal components of the BBB. ACs, whose end feet cover nearly the entire surface of BECs, have a critical effect on the BBB (Cheslow and Alvarez, 2016). Various AC proteins such as aquaporin-4 (Aqp4) and the Kir4.1 potassium channel localize to the end-foot membrane to regulate water homeostasis (Engelhardt and Liebner, 2014; Figure 1A). ACs function in BBB maintenance and induce the barrier properties of BMECs through various pathways such as Wnt (Guérit et al., 2021), Shh (Xing et al., 2020), and VE-PTP-dependent restoration of Tie2 signaling (Gurnik et al., 2016). The interaction between ACs and BMECs not only improves AC differentiation *via* the secretion of leukemia inhibitory factor 1 (LIF1) by BMECs but also maintains the development of the BBB through VE-PTP-dependent restoration of Tie2 signaling (Liebner et al., 2018). However, the relationship between PCs and ACs remains undetermined (Figure 1A).

## Effects of inflammation on blood–brain barrier

**FIGURE 2:**
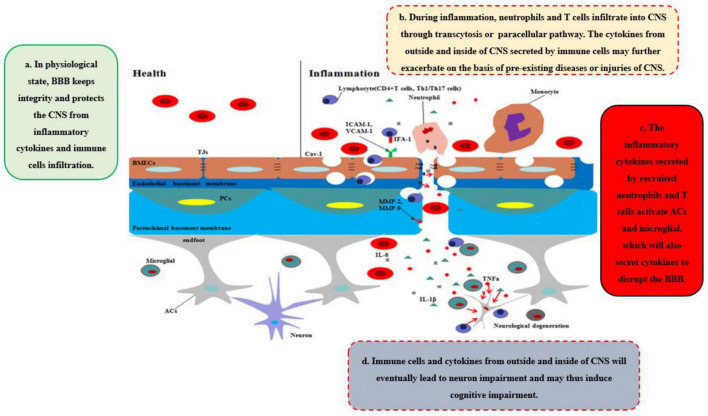
Disruption of BBB recruits the immune cells and cytokines into CNS. In healthy status, the integrity of BBB protects the CNS from toxins, pathogens, inflammatory cytokines, and immune cell infiltration. However, during inflammation, neutrophils and T cells from outside of CNS are recruited into CNS through transcytosis or paracellular pathway, which secrete pro-inflammatory cytokines and chemokines to further disrupt the integrity of BBB and deteriorate the CNS disease or injuries. Moreover, ACs and microglia are further activated by the cytokines, chemokines, and immune cells outside of CNS, which integrated with inflammatory factors outside of CNS will eventually lead to neurodegeneration and may induce cognitive impairment.

During neurological diseases or injuries, immune cells inside the brain secrete pro-inflammatory cytokines, and the structure of BBB tends to be loosened, including the disruption of PCs, which regulate the transcytosis, and TJs, which regulate the paracellular pathway (Figure 1C). Immune cells from outside the brain, such as T cells, specifically target adhesion molecules on BBB, particularly intercellular adhesion molecule-1 (ICAM-1) and vascular cell adhesion molecule-1 (VCAM-1) (Carrano et al., 2012). Subsequently, immune cells, especially CD4 + T cells, pass through BMECs and into the CNS *via* the transcytosis, mediated by caveolae cluster adhesion molecules, and through the matrix membrane *via* matrix metalloproteins (MMPs) expression by neutrophils in the early stage. The cytokines secreted by immune cells disrupt TJs to open the paracellular pathway in the late stage, which allows more immune cells to cross the BBB, especially Th1 and Th17 cells (Liebner et al., 2018). Immune cell reactivity and cytokine/chemokine secretion, therefore, play critical roles in the functional and anatomical structure of BBB and brain during neuroinflammation. However, our previous work suggested that diseases or traumatic injuries outside the brain may also induce BBB disruption and eventually lead to neurological degeneration through immune cells and pro-inflammatory cytokines (Yang et al., 2017; Yang J. et al., 2020). Both excessive activities can lead to BBB permeability and neurodegeneration, thereby worsening the progression of neurological diseases, such as AD. Given the ongoing debate regarding how immune cells gain entry into the CNS and induce BBB impairment and eventually neurological degeneration, we will systematically discuss the major cytokines (e.g., TNFα, IL-1β, and IL-6) that are secreted by immune cells and their impact on BBB and CNS (Figure 2).

### The effects of TNFα on blood–brain barrier and neurological degeneration

**FIGURE 3:**
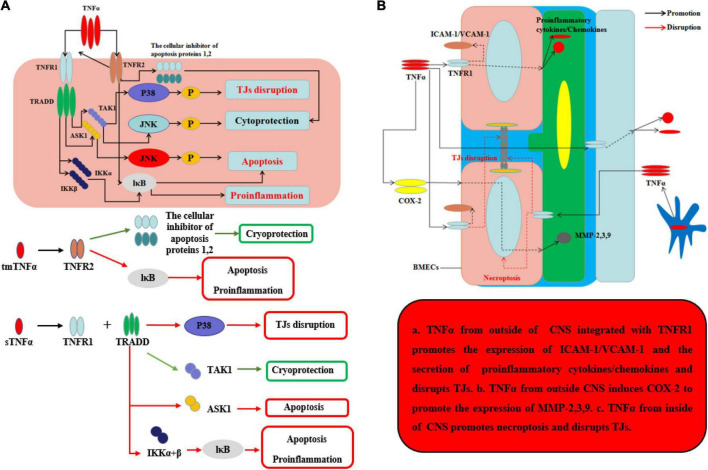
Effects of TNFα on BBB and neurological degeneration. **(A)** The mechanism of TNFα on CNS. The tmTNFα integrated with TNFR2 has two functions including promoting the expression of the cellular inhibitor of apoptosis proteins 1,2 to function as cryoprotection and integrating IκB to induce apoptosis and pro-inflammation. The sTNFα integrated with TNFR1 and TRADD can promote the expression of P38 to disrupt TJs and integrate with IKKα and β and further IκB to exert apoptosis and pro-inflammation. Moreover, the complex of sTNFα integrated with TNFR1 and TRADD can integrate TAK1 to exert cryoprotection through JNK in early stage, while the complex can integrate ASK1 to exert apoptosis through JNK in late stage. **(B)** The mechanism of TNFα on BBB from inside and outside of CNS. The sTNFα from outside CNS integrated with TNFR1 promotes the expression of ICAM-1/VCAM-1 on the membrane of BMECs and the secretion of pro-inflammatory cytokines and chemokines, which can disrupt the structure of TJs. Moreover, the complex of sTNFα with TNFR1 from outside CNS induces COX-2 to promote the expression of MMP-2,3,9 which can further disrupt the TJs and basement membrane. TNFα secreted mainly by ACs and microglia from inside CNS promotes necroptosis and disrupts TJs.

TNFα, the main homotrimeric transmembrane protein belonging to the TNF/TNFR ligand/receptor superfamily, plays an important role in neuroinflammation and BBB permeability (Table 1 and Figure 3). TNFα primarily acts by binding receptors on the cell surface as membrane-bound precursor (tmTNFα) and can also be secreted as a 51-kDa soluble trimer soluble TNFα (sTNFα) *via* proteolytic cleavage by the TNFα-converting enzyme (TACE) (Tang et al., 1996). During neuroinflammation, TNFα is secreted by neurons (Janelsins et al., 2008), astrocytes (Lieberman et al., 1989), and microglia (Janelsins et al., 2005) and interacts with its cognate receptors, TNF receptor type-1 (TNFR-1, also known as CD120a, p55/60) and TNF receptor type-2 (TNFR-2, also known as CD120b, p75/80). TNFR-1, which is expressed by almost all cells in CNS and mainly integrated with sTNFα, contains an intracellular death domain (DD) that plays an important role in TNFα–mediated apoptosis and cytotoxicity (Locksley et al., 2001; Sedger and McDermott, 2014). At the onset of neurological diseases or injuries, transient TNFα-induced JNK pathway plays a cytoprotective role through TAK1-dependent phosphorylation (Sato et al., 2005), while continuous TNFα-induced JNK signal leads to caspase-dependent apoptosis through JNK phosphorylation by apoptosis signal-regulating kinase 1 (ASK1) (Tobiume et al., 2001). In contrast, TNFR-2 plays an easy biological role compared to TNFR-1 and is mainly expressed by BMECs. Recent research has found that TNFα integrates with TNFR-2 can have effects on both pro-survival pathway and pro-inflammation through the activation of the cellular inhibitor of apoptosis proteins 1 and 2 and nuclear factor κB (NF-κB) (Rothe et al., 1995). Moreover, TNFR-2 can enhance the association with TNFR-1 and sTNFα to perform pro-inflammatory role in CNS (Tartaglia et al., 1993). Recent studies have found that TNFα inhibition TNFR-1–IgG administration was able to control the effects of experimental autoimmune encephalomyelitis (EAE) by reducing activated immune cells, including inflammatory leukocytes and microglia (Korner et al., 1997a). Korner et al. reported that mice lacking TNFα also developed EAE to the same extent as wild-type (WT) mice, because TNF^–/–^ mice were unable to form the typical mature perivascular cuffs observed in WT mice (Korner et al., 1997b). Neutralizing or knocking out TNFα has varying effects because the two types of TNFα receptor have different functions. It is the key that sustained sTNFα integrated with TNFR-1 plays a detrimental role in neurological diseases. To demonstrate this point, Suvannavejh et al. (2000) used TNFR-1/TNFR-2^–/–^ and TNFR-2^–/–^ mice and found that TNFR-1^–/–^ mice were able to control EAE progression by reducing Th1 cells and immune cell infiltration while TNFR-2^–/–^ mice have exacerbated the progression of EAE and the infiltration of immune cells (Suvannavejh et al., 2000). Sophie et al. found that a nanobody-based selective inhibitor of human TNFR1, TROS, was able to effectively delay disease onset, ameliorate symptoms, and prevent further disease development in a murine model of MS, in which the researchers generated mice expressing human TNFR1 in a mouse TNFR1-knockout background.

**TABLE 1:** Interaction between inflammatory cytokines and the BBB.

Inflammatory cytokines	Type	Effects on BBB
TNFα	TNFR-1, Pro-inflammatory effects	Disrupting TJs, especially Claudin-5 Chen et al., 2019
		Altered BBB morphology Ni et al., 2017
		Increased secretion of other pro-inflammatory cytokines Aslam et al., 2012
		Recruitment of leukocytes O’Carroll et al., 2015; Mantle and Lee, 2018
		Disrupting astrocytes van Kralingen et al., 2013
	TNFR-2, anti-inflammatory effects	Reduced recruitment of other pro-inflammatory cytokines Suvannavejh et al., 2000
		Promoting BBB recovery Madsen et al., 2020
IL-1β	Pro-inflammatory cytokine	Disrupting astrocyte
		Decreasing TEEB on the BMECs Argaw et al., 2006
		Increased secretion of other pro-inflammatory cytokines Labus et al., 2014
IL-6	sIL-6R, pro-inflammatory effects	Disrupting TJs Rosejohn and Heinrich, 1994
		Decreased β-catenin Yang et al., 2017
	mIL-6R, anti-inflammatory effects	Reduced recruitment of other pro-inflammatory cytokines
		Promoting BBB recovery Jones and Jenkins, 2018
HMGB1	Pro-inflammatory cytokine	Activating neuroinflammation
		BBB disruption O’Connor et al., 2003; Liesz et al., 2015
IL-10	Anti-inflammatory cytokine	Reduced BBB impairment
		Reduced down-regulation of Claudin-5 Couper et al., 2008; Lin et al., 2018

Under normal inflammatory condition, TNFα can promote BMEC remodeling through PC proliferation and differentiation from α1 to α2 integrin (Tigges et al., 2013). When neuroinflammation cannot be controlled, sustained TNFα can alter BBB permeability and make the neurological diseases or injuries worsen. During neurological diseases or injuries, microglia-induced TNFα interacts with TNFR-1 disrupts TJs and leads to BMECs necroptosis, thereby allowing entry of toxins and pathogens into CNS (Chen et al., 2019). Aslam et al. (2012) found that TNFα can disrupt the TJ structure by reducing Cldn5 *via* activation of the NF-κB signaling pathway. Ni et al. (2017) used an immortalized human cerebral endothelial cell line (hCMEC/D3) to demonstrate that TNFα can disrupt TJs on BMECs, which can be largely inhibited by SB202190, an inhibitor of p38MAPK, and partly by U0126, a MEK1/2-ERK1/2 inhibitor, thus implicating both of these signaling pathways in BBB disruption. From the outside of CNS, during systemic inflammation, peripheral TNFα can directly cause BBB dysfunction through decreasing transcellular electrical resistance, cellular polarity, and activate BMECs and ACs to secrete pro-inflammatory chemokines, such as MCP-1 and IP-10, which are responsible for the recruitment of leukocyte. The situation can further disrupt TJs and induce an inflammatory milieu, as well as the adhesion molecule ICAM-1 and VCAM-1, which recruit leukocytes by increasing the transcellular permeability of the BBB (O’Carroll et al., 2015; Mantle and Lee, 2018). In addition, Ding et al. suggested that TNFα can upregulate the expression of MMP-9, a proteinase to disrupt TJs and basal membrane of BBB, through activation of Ca/CAMK II/ERK/NF-κB signaling pathway (Ding et al., 2019). Other potential mechanism about peripheral TNFα-induced BBB dysfunction includes cyclooxygenase (COX) pathway and iNOS released. By activation of COX pathway, TNFα can upregulate COX-2 to increase the levels of MMP-2, MMP-3, and MMP-9 and change the cytoskeletal structure of BMECs to disrupt the TJs. Another pathway is TNFα-induced iNOS release which can increase the BBB permeability by impairing BMECs, but the mechanism remains unknown (Liu et al., 2013). At last, sustained TNFα in blood can lead to AC dysfunction. In other situations, hypoxia, recent studies indicate that TNFα will inhibit the expression of erythropoietin (EPO), which is secreted by ACs and has an important neuroprotective role on CNS and BBB, thereby exacerbating the outcome of neurological injury in hypoxia (Nagaya et al., 2014). Kralingen et al. found that TNFα generates an inflammatory milieu that will induce a reduction of ACs (van Kralingen et al., 2013). In contrast, although TNFα can induce BBB disruption and TNFα inhibition can reduce CNS inflammation, representing a potentially promising target for treating neuroinflammation, TNFα may play a neuroprotective role in diseases, such as AD, having been shown to reduce cellular prion protein (PrPC) and Aβ protein levels in BMECs of mouse. The effects of TNFα on the BBB and CNS are well known (Yasutaka et al., 2015). After all, the underlying intercellular signaling pathways, as well as strategies for using these effects to save patients from diseases, such as neurodegeneration, are promising targets for future study.

### The effects of IL-1β on blood–brain barrier and neurological degeneration

IL-1β, a member of the IL-1 family which includes seven agonist signaling ligands, three receptor antagonists, and IL-37, is largely responsible for the acute response and angiogenesis. During the initial inflammation, the inflammasome is synthesized in the cytoplasm which would assemble proteins made up mainly through the nucleotide binding and oligomerization domain (NOD)-like receptors (NLRs). This assembled inflammasome is specific for a set of cell damage-associated molecular patterns (DAMPs) or pathogen-associated microbial patterns (PAMP) according to the type of damage of body recognized by different NLR subtypes. Moreover, the inactive procaspase-1 molecules are recruited to the inflammasome complex to be activated (Nasti and Timares, 2012). IL-1β, primarily, occurs as a 31-kDa inactive precursor and would then be cleaved by the caspase-1 to yield the 17-kDa bioactive IL-1β (Netea et al., 2010). The low level of IL-1β in CNS performs an important biological and physiological role which controls sleep, appetite, hypothalamus–pituitary axis, neuroendocrine function (Liu and Quan, 2018), neurogenesis, and synaptic plasticity (Vezzani and Viviani, 2015). Furthermore, Goshen et al. found that blocking IL-1β signaling induced impaired hippocampal-independent memory and intact performance in adult mice (Cibelli et al., 2010). However, during neuroinflammation, sustained IL-1β integrated with toll-like receptor-4 (TLR-4) or IL-1 receptor (IL-1R) can promote the progression of diseases or injuries by activating ACs to secrete pro-inflammatory cytokines (IL-6, TNF), colony-stimulating factors, chemokines (CCL2, CXCL2, etc.), phospholipase A2, COX-2, prostaglandins and iNO *via* NF-κB and JNK pathways, and activator protein 1 (AP-1) (Blanco et al., 2005; Kitazawa et al., 2011; Musella et al., 2020). The activation of ACs can initiate microglia response to neuroinflammation by ACs-derived ATP as well (Bianco et al., 2005).

**FIGURE 4:**
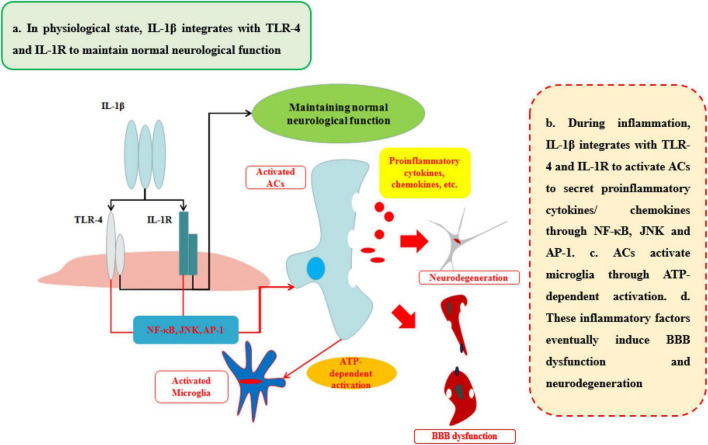
Effects of IL-1β on BBB and neurological degeneration. In healthy status, IL-1β integrates with TLR-4 and IL-1R to maintain normal neurological function in CNS. During inflammation, IL-1β integrates with TLR-4 and IL-1R to activate ACs to secrete pro-inflammatory cytokines and chemokines through NF-κB, JNK, and AP-1 pathways. In addition, ACs activate microglia through ATP-dependent activation. Finally, these inflammatory factors eventually induce BBB dysfunction and neurodegeneration.

The ability of IL-1β to increase BBB permeability after CNS injuries and neurological degeneration is well established (Table 1 and Figure 4). IL-1β disrupts BBB in two ways: First, IL-1β can activate ACs to disrupt BBB and exacerbate the progression of the neurological diseases or injuries. Recent work has shown that IL-1β induces the expression of hypoxia-inducible factor-1 (HIF-1) and its gene target, vascular endothelial growth factor-A (VEGF-A), in human astrocytes, which in turn induces the breakdown of BBB and exacerbates the CNS degeneration, such as in MS (Argaw et al., 2006). Moynagh et al. (1994) suggested that cerebral IL-1β can activate NF-κB and increase the levels of VCAM-1 and ICAM-1 in ACs, which can recruit the leukocytes into CNS. Second, IL-1β promotes the secretion of other pro-inflammatory cytokines (IL-6 and TNFα) to disrupt the paracellular BBB pathway. Labus et al. used a transfected human brain microvascular endothelial cell (THBMEC)-based *in vitro* BBB model and found that IL-1β decreased TEER and induced the secretion of other pro-inflammatory cytokines, including IL-6 and TNFα, to increase the paracellular permeability of BBB. The increasing paracellular permeability will be more vulnerable to leukocytes and thus exacerbate neuroinflammation (Labus et al., 2014). Some studies have shown that increasing IL-1β or TNFα has similar effects on the secretion of inflammatory cytokines and the induction of ACs death (Netea et al., 2010). Ni et al. found that IL-1β may be a downstream target of TNFα, which can change the structure of Ocln, thereby changing BBB permeability, a smaller effect than that of TNFα through p38 MAPK and ERK1/2 in hCMEC/D3 cells (Ni et al., 2017). However, these effects are quite different from the effects of TNFα, as demonstrated by O’Carroll et al. who observed clear differences between the effects of TNFα and IL-1β on BBB permeability. IL-1β preferentially induces the cytokines sICAM-1, IL-6, sTNFRI, sTNFRII, GCSF, and GMCSF and the chemokine IP10, whereas TNFα is more likely to induce RANTES and IL-8 secretion (O’Carroll et al., 2015). As a result, the sustained IL-1β will impair the BBB and then induce neurological degeneration. The fact makes the inhibition of IL-1β a therapeutic target for attenuating neuroinflammatory and neurodegeneration (Cibelli et al., 2010).

Despite the evidence of IL-1β on disruption of BBB and then inducing the progression of neurological diseases discussed above, there are supplements of the role of IL-1β induced by systematic inflammation. Mario et al. (2010) have found that mice undergoing surgery of the tibia under general anesthesia have an extreme increasement of IL-1β in hippocampal and then inducing memory and cognitive impairment (Mario et al., 2010). Our research indicated that burn disrupted BBB both increasing the paracellular pathway and transcytosis through increasing the peripheral level of IL-1β and IL-6 (Yang J. et al., 2020). However, the potential mechanism of how it infiltrates into CNS is still unclear.

### The effects of IL-6 on blood–brain barrier and neurological degeneration

**FIGURE 5:**
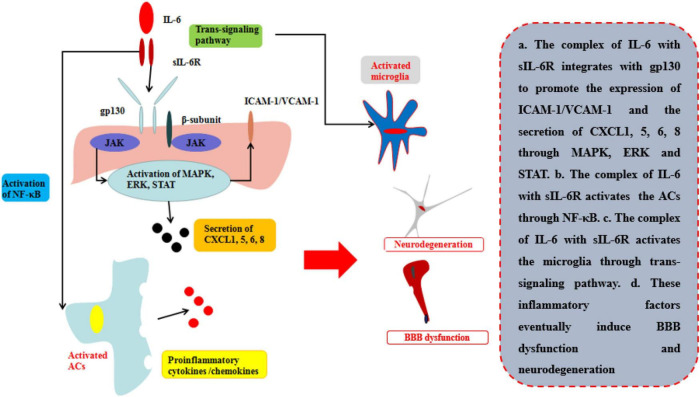
Effects of IL-6 on BBB and neurological degeneration. The complex of IL-6 with sIL-6R integrates with gp130 to promote the expression of ICAM-1 and VCAM-1 on BMECs and the secretion of CXCL1, 5, 6, 8 through MAPK, ERK, and STAT. Moreover, the complex of IL-6 with sIL-6R activates the ACs through NF-κB pathway to secrete pro-inflammatory cytokines and the microglia through trans-signaling pathway in CNS. Finally, these inflammatory factors eventually induce BBB dysfunction and neurodegeneration.

The common receptor to bind IL-6 is receptor β-subunit, membrane glycoprotein 130 (gp130; also known as IL-6Rβ), which works with either a non-signaling receptor α-subunit or a signaling receptor β-subunit like gp130 to exert pleiotropy function (Heinrich et al., 2003). In addition to classical signaling pathway binding with gp130 on membrane, there is a soluble receptor mainly associated with IL-6(sIL-6R) which can bind circulating half-life IL-6 and expand its bioactivity in those cells without gp130 on their membrane. This signaling pathway is called trans-signaling pathway and is involved in numerous chronic inflammation and diseases (Rosejohn and Heinrich, 1994) (Table 1; Figure 5). During neuroinflammation, normal level and biological function of IL-6 interacted with sIL-6R *via* trans-signaling pathway can induce repopulating microglia which has an important role on neurogenesis specifically by augmenting the survival of newborn neurons that directly support cognitive function (Willis et al., 2020). The level of IL-6 increases by the activation of ACs induced by TNF and IL-1β *via* NF-κB signaling pathway, while IL-6 is negatively regulated by Wnt signaling pathway (Edara et al., 2020). However, other research indicates that sustained IL-6 can lead to neuroinflammation and pain post-injury *via* signal transducer and activator of transcription 3 (STAT3) (Hu et al., 2020). Through STAT3, IL-6 can induce the demethylation of NeuroD1 (neurogenic differentiation 1) in neural stem cells (NSCs) to promote the neurogenesis in AD as well (Kong et al., 2019). Moreover, Escrig et al. found in mouse AD model that IL-6 trans-signaling induced increasing levels of amyloid plaques and neurofibrillary tangles and cognitive dysfunction (Escrig et al., 2019).

Recent research indicates that the peripheral levels of IL-6 are higher compared with those healthy people in those patients with neurological diseases or injuries (Robelin and Gonzalez De Aguilar, 2014; Yang et al., 2017; Yang J. et al., 2020). The disruption of BBB is mainly by the integration of complexes of sIL-6R and gp130 on the membrane of BMECs by trans-signaling pathway. The pathway can directly disrupt the TJs of BBB in one respect and indirectly increase the ICAM-1 and VCAM-1 to recruit neutrophils infiltrating into the inflammatory site in another. Rosejohn and Heinrich (1994) have found that blockage of the trans-signaling pathway can effectively reduce the leakage of BBB and then relieve the progression of neurological degeneration. As mentioned of TNFα and IL-1β, IL-6 can also induce the released pro-inflammatory cytokines/chemokines to deteriorate the progression in several neurological diseases or injuries (Mantle and Lee, 2018). Zhang et al. (2015) found that peripheral IL-6 levels were increased during hypoxic-ischemic brain injury, thus disrupting BBB permeability, and that using neutralizing anti-IL-6 antibodies (anti-IL-6AB) decreased the disruption of TJs and controlled BBB permeability 24 h after ischemic injury. Emery et al. suggested that using an inhibitor of IL-6R can effectively control the progression of autoimmune diseases, such as amyotrophic lateral sclerosis (ALS), in a phase-three clinical trial (Emery et al., 2008).

Above all, most researches focus on the evidence that the inflammation caused by CNS diseases or injuries can induce neurodegeneration. However, peripheral IL-6 can also induce the BBB dysfunction. Our work using a murine surgery model revealed that surgical wounds can induce age-associated BBB dysfunction, and elevated serum IL-6 levels led to decreased TJs but had no effects on AJs (Yang et al., 2017). In addition, we used the mouse burn model which suggested that burn can increase the peripheral level of IL-6 and IL-1β and the level of MMP-9 to disrupt the integrity of BBB *via* paracellular pathway by decreasing TJs, and transcytosis by decreasing Mfsd2a (Yang J. et al., 2020). Other research indicates that the increasing peripheral level of IL-6 can disrupt the TJs to disrupt the BBB and cause neuroinflammation on one side (Furutama et al., 2020) and induce the increasing level of COX-2 to disrupt the BMECs (Eskilsson et al., 2014). Our previous study also found that increased peripheral IL-6 levels can induce decreased β-catenin levels, thereby activating the Wnt pathway, suggesting that the ability of IL-6 overexpression to disrupt the BBB may be related to Wnt signaling pathway (Yang et al., 2017). The study will provide a new insight how we should prevent the CNS injuries from the other site injuries or diseases.

### The effects of other potential cytokines on the blood–brain barrier and neurological degeneration

Inflammation is controlled by a complex network of various pro-inflammatory cytokines and receptors (Table 1; Patin et al., 2016). However, the exact cytokines and receptors that participate in the neuroinflammation and how they produce their effects *via* signaling pathways remain unclear. In this section, we will try to elucidate the current possible factors and signaling pathways involved in BBB disruption. High-mobility group box 1 protein (HMGB1), which belongs to the damage-associated molecular pattern (DAMP) family (Chaudhry et al., 2018), is released from stressed and dying brain cells and is a potent neuroinflammatory mediator that is mainly regulated by post-translational redox modification. HMGB1 is a nuclear DNA-binding protein that contains two DNA-binding domains (boxes A and B) and a highly repetitive, negatively charged C-terminal tail (Thundyil and Lim, 2015). HMGB1 can disrupt the BBB and induce neurodegeneration by promoting the secretion of pro-inflammatory cytokines, such as TNFα, IL-1β, and IL-6, through the NF-κB signaling pathway and by activating the RAGE axis (O’Connor et al., 2003; Liesz et al., 2015). Moreover, intentionally blocking HMGB1 may improve the outcomes of neurological diseases and injuries such as stroke and TBI and protect the BBB from disruption (Zhang et al., 2011; Okuma et al., 2014).

In contrast, some cytokines play an important role on anti-inflammation and protect the permeability of BBB, including interleukin-10 (IL-10), which is an anti-inflammatory cytokine that is expressed by various leukocytes. The two main IL-10 receptors are IL-10Rα, which is specific to IL-10, and IL-10Rβ (Sanjabi et al., 2009). IL-10 mainly exerts its function by activating the STAT3 signaling pathway. The IL-10 gene promoter contains a STAT3 binding site, resulting in a feedback loop for this pathway. Interestingly, STAT3 plays an anti-inflammatory role when it is activated by IL-10 but plays a pro-inflammatory role with IL-6 (Mosser and Zhang, 2008). Regarding its effects on the BBB and CNS, Lin et al. suggested that IL-10 effectively reduced BMEC apoptosis induced by IL-6-activated STAT3 signaling during neuroinflammation and ameliorate the downregulation of Cldn5 expression in the BBB in a rat model, while *in vitro*, IL-10 protected BBB integrity against TNFα (Lin et al., 2018). Overall, IL-10 plays an important role in immune system homeostasis. Reduced IL-10 expression increases susceptibility to autoimmune inflammation, whereas IL-10 overexpression will impede the clearance of pathogens and induce a chronic inflammatory state (Couper et al., 2008). As a result, how to use IL-10 for clinical treatment requires further study.

In addition to major cytokines, such as TNFα, IL-1β, IL-6, and their receptors, other cytokines also play an important role in neuroinflammation and neurodegeneration and may provide some promising new targets for clinical application to treat neurological disease and injury. However, determining how these factors interact with each other and how the mechanisms of these effects change with age is of critical importance.

## The effects of inflammatory cytokines on blood–brain barrier and central nervous system from primary neurological diseases or systematic inflammation

In primary neurological diseases including AD, MS, stroke, and TBI, inflammatory cytokines play an important role on the promotion of the diseases healing or deteriorating the progression of the diseases in early or late stage, respectively. Rational regulation of inflammation will be the protection for the primary CNS diseases (Jayaraj et al., 2019). However, in neurological diseases, damage to the CNS may lead to a decrease in the control of inflammation, which will lead to inflammatory dysregulation (Lambertsen et al., 2019; Ren et al., 2020). Under this condition, the aberrant expression of inflammatory cytokines will induce the deterioration of neurological diseases and thus neurodegeneration, which may provide an effective target for future clinical treatment as well. Neurological diseases like AD, the secondary pro-inflammatory insults, trigger delirium and can accelerate cognitive decline. Lopez-Rodriguez et al. (2021) found that Aβ was surrounded with NLRP3-integrated inflammasome which recruited microglia to be primed and facilitated to secrete IL-1β using APP/PS1 mice. In addition, activated ACs were primed to pro-inflammatory chemokine responses induced by IL-1β. A CNS trauma like TBI (Kuwar et al., 2019) also found that the complexes of NLRP3-integrated inflammasome induced the expression of IL-1β which will subsequently induce the expression of TNFα and IL-6. On the other side, those pro-inflammatory cytokines secreted by the microglia and ACs can further induce the disruption of BBB through the pathway mentioned before. Furthermore, the expression of ICAM-1 and VCAM-1 and the disruption of TJs will subsequently recruit the peripheral immune cells and cytokines into CNS and thus accelerate the progression of primary diseases (Moynagh et al., 1994; Argaw et al., 2006; Labus et al., 2014; O’Carroll et al., 2015; Zhang et al., 2015; Mantle and Lee, 2018).

Nowadays, the CNS injury caused by peripheral trauma or surgery has gradually become a research hotspot which could also trigger delirium and accelerate cognitive decline on the basis of pre-existing diseases or with higher underlying risks of neurological diseases (Yang T. et al., 2020). Our research focused on the disruption of BBB and cognitive impairment induced by peripheral traumatic injuries. We used traumatic surgical model and burns model in mice and found that peripheral inflammatory cytokines, including IL-6 and IL-1β, were main factor leading to the disruption of BBB and thus cognitive impairment. In surgical model, we found that BBB was disrupted by the increasing level of IL-6 and thus induced cognitive impairment, especially in old mice with higher serum level of IL-6 than young mice with that (Yang et al., 2017). To fully understand the mechanism about the disruption of BBB induced by systematic inflammation, we further used burns model which had more serious inflammatory response than our previous study used surgical model. We found that the increasing permeability of BBB not only included paracellular pathway but also transcytosis induced by the increasing serum level of IL-1β and IL-6 (Yang J. et al., 2020). Unlike primary CNS disease, there are few inflammatory-mediated factors to specifically recruit the immune cells or inflammatory cytokines into CNS. From our studies, the potential mechanism about the disruption of BBB and thus CNS impairment induced by systematic inflammation may be caused by high amount and functional degeneration of immune cells and pro-inflammatory cytokines to identify the receptors in BMECs, the immune situation of which is more likely occur in aging or chronic diseases population (Sendama, 2020). However, new research associated with AD mentioned a potential mechanism that peripheral surgery inducing cognitive impairment may be through the disruption of nasal epithelium and olfactory receptor neurons by the increasing level of IL-6 after surgery (Zhang et al., 2022), but the sequence of disruption of nasal epithelium, olfactory receptor neurons, and cognitive impairment was not strictly discovered, which indicated that peripheral trauma inducing cognitive impairment through the disruption of BBB caused by systematic inflammation may still be the most probable theory in the field. Cognitive impairment and then neurological degeneration induced by systematic inflammation are our novel insight, which will need to pay attention to the protection of BBB in clinical treatment of peripheral diseases in future. However, the potential mechanism needs to be further studied.

## Conclusion

The primary factors that disrupt the BBB to induce neurological degeneration are three inflammatory cytokines: TNFα, IL-1β, and IL-6. The basal functions of these cytokines are elucidated above. As an inflammatory network, these cytokines integrated with their special receptors can similarly disrupt TJs and TEER of the BBB without any impairment of AJs (Aslam et al., 2012; Labus et al., 2014), promote ACs apoptosis (Argaw et al., 2006; Nagaya et al., 2014), and recruit leukocytes into BBB (O’Carroll et al., 2015), thereby directly or indirectly inducing neurological degeneration. Other cytokines, including HMGB1 and IL-10, also play an important role in the BBB, as mentioned above. Cytokines such as TNFα and IL-6 bind their respective receptors and exert different functions, as discussed above (Locksley et al., 2001; McCoy and Tansey, 2008; Murakami and Hirano, 2012; Sedger and McDermott, 2014; Wolf et al., 2014). Currently, the pathways that may be involved in these processes include NF-κB (Bianco et al., 2005; Aslam et al., 2012; Edara et al., 2020), p38MAPK, and MEK1/2-ERK1/2 signaling pathway (Ni et al., 2017). However, the potential mechanisms by which cytokines attack the BBB are still unclear. Recent work has shown that blocking pro-inflammatory cytokines, including TNFα, IL-1β, and IL-6, can reduce the permeability of BBB and improve the outcomes of neurological diseases and injuries to a great extent. However, in some conditions, studies have found that cytokines such as TNFα, IL-1β, and IL-6 can also improve the outcome of diseases such as ADs and that neutralizing or knocking out these cytokines can eventually attenuate the progression of neurological diseases or injuries (Goshen et al., 2007; Yasutaka et al., 2015; Han et al., 2016; Patin et al., 2016). Overall, neurological diseases or injuries present a substantial challenge to the medical field because they are difficult to diagnose and cure and are a social burden for families. Almost all neurological diseases and injuries have an important relationship with the BBB and inflammatory cytokines, which represent a new target for treating these diseases and injuries. As a result, the effects of these cytokines on the BBB and neurodegeneration must be investigated to develop new clinical treatments.

The most recent research has focused on local CNS diseases or injuries such as TBI, AD, and MS attacking BBB and inducing neurodegeneration (Owen et al., 2010; Vázquez-Rosa et al., 2020; Puthenparampil et al., 2021). However, our previous research has confirmed that systematic inflammation will also induce neurological degeneration. For example, after peripheral surgery, the concentration of IL-6 increases in peripheral blood and the structure of the BBB breakdown, thereby inducing cognitive impairment in mice. Older individuals (18-month-old mice) have a higher risk of such impairment. Using IL-6-neutralizing antibodies or gene IL-6 knockout protected the BBB from disruption (Yang et al., 2017). Furthermore, we use the mouse burn model to indicate that peripheral burn can also increase the level of IL-6 and IL-1β in serum which disrupt the integrity of BBB through paracellular pathway and transcytosis (Yang J. et al., 2020). Similar results have been observed in humans. Reichenberg et al. (2001) found that humans with Salmonella abortus equi endotoxin (0.8 ng/kg) presented with abnormal behavior compared with healthy individuals. The results revealed that even a small amount of inflammation can impair the BBB and induce cognitive impairments. As a result, with advancing age, people are more vulnerable to various diseases, and their body experiences chronic inflammation. This effect may continue to produce pro-inflammatory cytokines that disrupt the BBB and induce neurological degeneration. However, demonstrating these effects and illuminating the detailed mechanisms require substantial future research.

Current treatments for neurological degeneration are minimally effective. To date, most studies have found that inhibiting the overexpression of inflammatory cytokines is effective at improving the outcomes of neurological diseases and injuries and delaying the onset of neurodegeneration. However, the timing and mode of intervention are not ideal. As in our research, using anti-IL-6 antibody 18 h before surgery effectively reduced BBB disruption and cognitive impairment, whereas post-surgery treatment has no effect. In clinical settings, we cannot readily use protective interventions before injuries or diseases. Additional research has also found that mesenchymal stem cells (MSCs) effectively protect BBB integrity and that MSC intracranial transplantation may be a promising method for future treatment (Si et al., 2011). Tang et al. found that MSC intracranial transplantation protected BBB integrity, inhibited ACs apoptosis, and decreased Aqp4 expression, which reduced brain edema and impairment (Tang et al., 2014). Menge et al. (2012) suggested that the injection of MSCs can increase the level of tissue inhibitor of matrix metalloproteinase-3 (TIMP3) which can inhibit VEGF-A to protect the BBB from disruption. As mentioned before, injecting MSCs requires cerebral transplantation, which involves substantial risk, and the exact mechanism of how MSCs influence the BBB is poorly understood. Our previous study suggested that MSCs injecting through the tail of mice can effectively protect the integrity of BBB by decreasing peripheral inflammatory cytokines including IL-1β and IL-6, the level of MMP-9, and increasing the level of Mfsd2a (Yang J. et al., 2020). However, the mechanism by which MSCs prevent the BBB from pro-inflammatory cytokines attacking is still under discussed. As a result, additional studies are needed before this approach can be used in clinically.

Individuals with acute or chronic non-cerebral diseases account for a large proportion of patients in the world, and few treatments are available to protect the BBB. Especially, aging makes BBB more vulnerable to the disruption by inflammatory cytokines. The impairment of BBB is easy to recruit more immune cells and cytokines into brain parenchyma and then induce neurological degeneration (Boland et al., 2018). With an aging society coming, the medical care of aging people with neurological diseases is commonly considered a significant economic burden on society and on families (Chen, 2010; Gonneaud and Chételat, 2018; Fang et al., 2020; Pandya and Patani, 2021). As a result, whether the prevention of the disruption of BBB from non-cerebral injuries and diseases will delay the outset and improve the progression of neurological degeneration and this question requires urgent and massive future research to approve. As such, future studies should focus on the mechanisms and treatment of peripheral inflammation-induced BBB disruption. Regardless of cerebral or non-cerebral inflammation, protecting the BBB is a key to preventing neurological degeneration.

## Author contributions

SY, JY, MR, YL, and XF conceived and designed the review. JY, YL, KM, and YY prepared the figures. JY, HL, SY, and XF wrote the manuscript. All authors contributed to the article and approved the submitted version.

## Glossary

AD: Alzheimer’s disease
MS: multiple sclerosis
TBI: post-traumatic brain injury
BBB: blood–brain barrier
CNS: central nervous system
NSCs: neural stem cells
TNFα: tumor necrosis factor α
IL-1β: interleukin-1beta
IL-6: interleukin-6
iNOS: inducible nitric oxide synthase
BECs: brain endothelial cells
PCs: pericytes
ACs: astrocytes
TJs: tight junctions
AJs: adherens junctions
Cldn5: claudin-5
Ocln: occludin
ZO: zonula occludens
VE: vascular endothelial
Aqp4: aquaporin-4
Kir4.1: inwardly rectifying 4.1
shh: sonic hedgehog
VE-PTP: vascular endothelial protein tyrosine phosphatase
Tie2: type I tyrosine kinase receptor 2
ECs: endothelial cells
ICAM-1: intercellular adhesion molecule-1
VCAM-1: vascular cell adhesion molecule-1
MMPs: matrix metalloproteinases
PD: Parkinson’s disease
TACE: TNFα-converting enzyme
TNFR: tumor necrosis factor receptor
DD: death domain
NF-κB: nuclear factor κB
EAE: experimental autoimmune encephalomyelitis
WT: wild-type
hCMEC/D3: immortalized human cerebral endothelial cell line
EPO: erythropoietin
PrPC: cellular prion protein
MBEC4: mouse brain microvascular endothelial cell line
IL-37: interleukin-37
HIF-1: hypoxia-inducible factor-1
VEGF-A: vascular endothelial growth factor-A
THBMEC: transfected human brain microvascular endothelial cell
TEER: transendothelial electrical resistance
IL-11: interleukin-11
IL-27: interleukin-27
IL-31: interleukin-31
OSM: oncostatin M
LIF: leukemia inhibitory factor
CNTF: ciliary neurotrophic factor
CT-1: cardiotrophin 1
CLCF1: cardiotrophin-like cytokine factor 1
gp130: membrane glycoprotein 130
CXCL: CXC-chemokine ligand
HMGB1: high-mobility group box 1 protein
DAMP: damage-associated molecular pattern
MHC: major histocompatibility complex
RAGE: receptor for advanced glycation endproducts
STAT3: signal transducers and activators of transcription
MAPK: mitogen-activated protein kinase
IL-10: interleukin-10
MSCs: mesenchymal stem cells.
